# Supplementary material for: Adolescent Body Dissatisfaction in Contrasting Socioeconomic Milieus, Coming from a French and Luxembourgish Context
Source: Int J Environ Res Public Health. 2019 Dec 20;17(1):61. doi: 10.3390/ijerph17010061 (PMC6982110; doi:10.3390/ijerph17010061)
Supplement: Supplementary file 1 [file ijerph-17-00061-s001.pdf]

APPENDIX 1

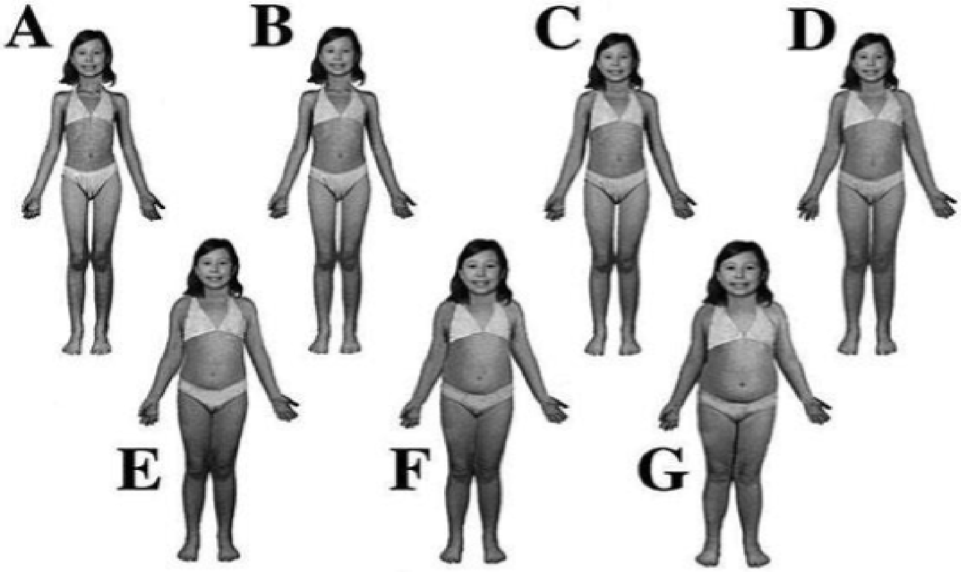

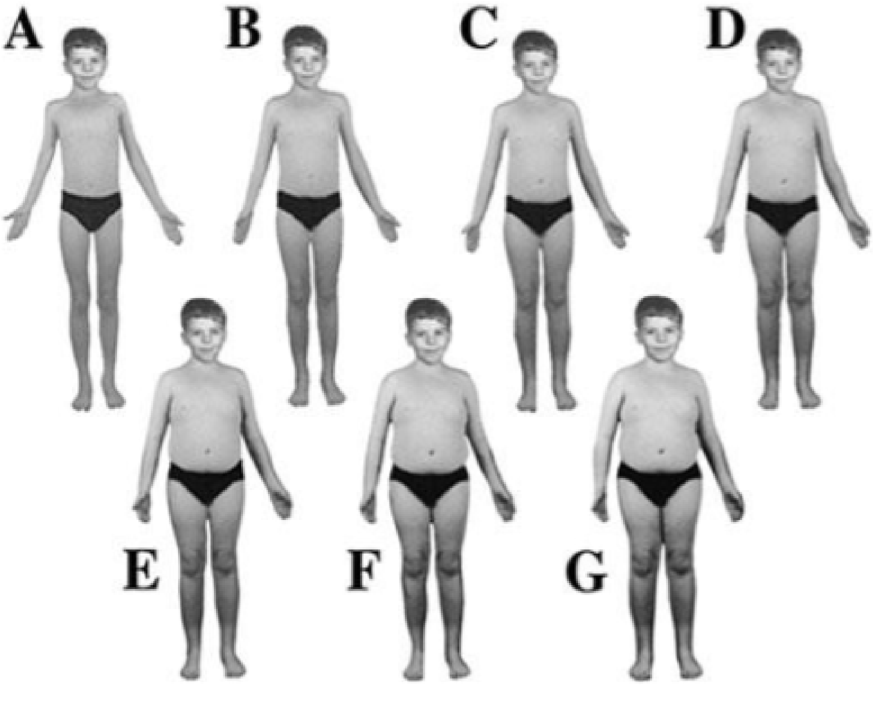

Figure 1: Pictures presented to the girls and the boys of the Children's Body Image Scale Children [10]
